# Using Illicit Drugs to Lose Weight among Recovering Female Drug Users in China: An Exploratory Qualitative Study

**DOI:** 10.3390/ijerph19052626

**Published:** 2022-02-24

**Authors:** Liu Liu, Xiaotao Wang, Yang Xie, Wing-Hong Chui

**Affiliations:** 1 School of Social and Behavioral Sciences, Nanjing University, Nanjing 210023, China; 2 School of Social Development, Nanjing Normal University, Nanjing 210097, China; chipie@njnu.edu.cn; 3 Faculty of Education, Beijing Normal University, Beijing 100875, China; xieyang@mail.bnu.edu.cn; 4 Department of Applied Social Sciences, The Hong Kong Polytechnic University, Hong Kong, China; wing-hong.chui@polyu.edu.hk

**Keywords:** weight loss, illicit drug use, qualitative method, China

## Abstract

The population of female drug users has been growing in China, and these women have been found to care deeply about their weight. Against this backdrop, this study examines the relationship between Chinese women’s illicit drug use and their intentions to lose weight, keep fit, and maintain a slim body shape. The participants of this study were 29 women who all had experience with illicit drug use for weight control. These women were drawn from a female compulsory drug treatment center located in eastern China. Semi-structured interviews with these 29 participants were conducted between 2013 and 2016. Expectations of losing weight and pursuing their ideal slim body shape were found to be an important reason for the study participants’ initiation of drug use, its maintenance, and failures to achieve abstinence. These Chinese female drug users were generally satisfied with weight loss outcomes subsequent to drug consumption. A fuller appreciation of Chinese women’s weight-loss-related illicit drug use patterns is much needed to help devise strategies and policies to deal with this growing problem. These include changing the dominant aesthetic cultural preference for thinness, paying particular attention to the functional use of illicit drugs in drug treatment programs, and having special interventions for women who interact with drug users within their social networks.

## 1. Introduction

People’s perceptions of an ideal body weight and shape are influenced by social and cultural factors, such as gender differences, health concerns, and religion, and, thus, differ across cultures and have differed over time [1,2,3,4]. Certain developing countries value full body figures, since these represent wealth, abundance, and fertility [5]. This perception was also common in North America and some European countries prior to 1900 for the same reason [6]. However, today, in developed countries where food availability is plentiful and good health is valued, larger body sizes are no longer considered desirable; instead, thinness has become fashionable [1]. Globalization has encouraged this aesthetic standard to spread to developing countries hand in hand with urbanization, modernization, and westernization [7].

Women are more likely to internalize the sociocultural ideals associated with body shape portrayed in the globalized media [8]. Women tend to be much less satisfied with their bodies, see themselves as overweight, and engage in extreme weight loss practices, although most are actually within a healthy weight range [9,10,11]. In addition, far stronger relationships between body shape dissatisfaction and comparisons with others are found among women than men, which indicates that women’s attitudes towards their bodies are more easily influenced by social evaluation and pressure [8,12]. Furthermore, women are more likely to pursue an extremely thin body shape in order to conform to men’s perceptions of female attractiveness [8].

In order to become or remain slim, many women, particularly those in their adolescence and young adulthood, are at high risk of engaging in dysfunctional weight loss and weight control practices. These practices include not merely disordered eating [7,10], but also substance use [13]. Prior research has shown that there is a significant positive relationship between female adolescents’ substance use behaviors (including the use of alcohol, cigarettes, and illicit drugs such as marijuana and cocaine) and weight control attitudes and activities [14,15]. Body perception and weight loss expectations have been found to impact heavily on women’s illicit drug use [16,17,18,19].

While existing research has revealed the link between women’s weight control practices and their illicit drug use behaviors, most has focused on the negative consequences of the drug use behavior and regards drug use as “dysfunctional” in terms of achieving the goal of weight loss [20,21]. However, when female users believe that illicit drug use is an acceptable or even ideal way to control weight, they engage in drug use motivated by what they perceive as a positive outcome in mind. Therefore, in actuality, female drug users’ subjective understandings of and attitude towards illicit drugs as tools for weight management drives their drug use. Few studies have addressed female drug users’ individual ideas regarding the functional aspects of illicit drugs, and even fewer in relation to weight control.

After China’s vast social changes during the twentieth century, and rapid globalization more recently, seeking thinness is becoming the most prevalent attitude towards body image in the country [22,23]. At the same time, China is also experiencing rapid growth in its population of drug users, especially young female users [24]. We observed through our ongoing research into young people’s substance use attitudes and behaviors that some of the young women concerned are experimenting with substances (including cigarettes, e-cigarettes, and illicit drugs) to manage their weight. Studies of illicit drug use in China have proliferated in recent years [25], but research centering on women’s illicit drug use in relation to weight loss and weight control is still scarce. To the best of our knowledge, there are hardly any studies of Chinese female drug users’ perspectives on illicit drugs as a means of weight control. To help fill this void, this study aims to explore the relationship between Chinese women’s use of illicit drugs and their thoughts on weight loss, keeping fit, and maintaining a slim body shape. Moreover, instead of only focusing on the initiation of drug consumption, this study plans to interrogate Chinese female drug users’ perceived need for weight control throughout the drug abuse career [26].

## 2. Materials and Methods

### 2.1. Participants

As an exploratory study, a qualitative method was adopted to collect and analyze data. More specifically, thematic analysis, a commonly used qualitative approach, was employed to identify themes and subthemes related to illicit drug use and weight control in experiences solicited from interviews with a small-sized convenience sample of 29 female drug users [27,28,29]. The participants of this specific study were part of a larger pool of participants in a project on Chinese drug users’ lives.

The larger project collected both qualitative and quantitative data through several rounds of interviews and questionnaire surveys with both female and male drug users living in different compulsory drug treatment centers between 2013 and 2016. A compulsory drug treatment center in China is a facility used to provide institutional treatment for individuals convicted of illicit drug use three or more times [30]. It is not a prison, with facilities and programs tailored to drug treatment, but residents there cannot leave before the end of the treatment. Individuals in these centers are usually considered drug addicts [28]. The project was conducted according to the guidelines of the Declaration of Helsinki and approved by the Institutional Review Board of the School of Social and Behavioral Sciences at Nanjing University. In order to protect the human rights of the participants, the administrative boards of all the compulsory drug treatment centers concerned also read and accepted the research protocol of the project prior to data collection.

During the qualitative data collection period of the wider project, all female drug users were reached in two female compulsory drug treatment centers, one in eastern and one in western China. They were recruited based on the criteria of maximum variation [31], on a voluntary basis, and with recommendations of suitable participants by the centers’ administrative officers. Adopting a purposive sampling strategy, the research team first asked the administrative officers to help invite female drug users with diverse drug use experiences and demographic backgrounds to participate in the project. These potential participants were fully informed of the aims and also the privacy protection protocols of the project before participation. They had right to freely choose participation or not, without any negative consequences. Ultimately, 77 women with different backgrounds and drug use histories participated in interviews, and 4 women declined. Participants all signed informed consent documentation before their participation.

In accordance with the study’s aim, the participants included only those female drug users who had used illicit drugs for weight control. Among the 77 women, 29 shared their experiences of weight-loss-related illicit drug use and, thus, formed the final sample for this specific study. Coincidently, these 29 participants were all from one center located in eastern China and with around 1200 female drug users.

The demographic and drug use information of the 29 participants is outlined in Table 1. These female illicit drug users’ average age was 31.0, with 16 being the youngest and 48 the eldest. Seventeen of these women had received nine years of education or less. Only one participant had junior college experience. Twenty-three participants reported that they were single (including divorced and widowed) when mentioning their marital status. Among all participants, 13 women had legal full-time jobs before entering the compulsory drug treatment center, 7 worked in the (illegal) sex industry, 4 were self-employed or running small businesses, and the other 5 were unemployed. Fourteen participants were exclusively methamphetamine users, another fourteen reported that they were polydrug users and most of them used heroin together with methamphetamine, and only one woman identified as a user of heroin exclusively.

### 2.2. Data Collection and Analysis

Chinese female drug users’ illicit drug use experiences that related to weight loss were collected through semi-structured, one-on-one interviews [32]. Several open-ended questions were asked during the interviews, such as “Could you please describe your experiences of using drugs for weight loss?”, “How did you know that using drugs is a way to facilitate weight loss?”, “Why did you decide to use drugs to lose weight?”, “Do you think you can relinquish drug use after treatment?”, and “What is your opinion of using drugs for weight control?”. The participants were encouraged to tell their own stories and express their opinions during the interviews. Set questions and spontaneous follow-up questions were asked based on interviewees’ stories and responses to learn the details and uniqueness of each interviewee’s experiences. Using Mandarin Chinese as the communication language, all interviews were audio recorded and lasted between 60 and 90 min.

Eight research assistants trained in interviewing techniques served as the interviewers for the whole project, including for these 29 interviews. These assistants were all post-graduate students in the School of Social and Behavioral Sciences at Nanjing University, and had learned and practiced interviews prior to the implementation of the project. They had not met any of the participants prior to the interviews. To ensure the quality of the interview data, the first author also provided all interviewers with both training beforehand and on-going supervision. Specific training and supervision focused not only on appropriate interviewing strategies, but also making interviewers aware of the possible social reasons influencing individuals’ drug use; thus, minimizing their possible negative views of drug users as well as their drug use behaviors [28]. As such, interviewers could generally show respect to the participants and, thus, offer friendly conversation for successful interviews.

The 29 interviews were transcribed verbatim by the above mentioned eight research assistants after they had finished the interviews, and then analyzed based on the thematic analysis guidelines suggested by Braun and Clarke [27]. The first and second authors formed a team to analyze the data, as both authors are professional social scientists with qualitative analytical skills and understanding of drug use and drug users. To be familiar with and better interpret the data, the analysis started with a close, line-by-line reading of the transcripts. Several initial codes that related to the aim of the study were identified during this process. After generating these initial codes, mind maps were used to inductively categorize the initial-coded data under different candidate themes and sub-themes. After reviewing, discussing, and refining the candidate themes and sub-themes, three main themes and related sub-themes were finally identified and were presented in the next section. Through this process of thematic analysis, Chinese female drug users’ weight-loss-related illicit drug use experiences were organized into a concise yet informative picture.

All participants’ private information was kept confidential in both data collection and analysis. We used pseudonyms to replace the participants’ real names and ensured the identities of individual participants would not be revealed through combinations of their quotes and descriptions.

## 3. Results

The results below present the experiences of weight loss among Chinese female drug users according to three key themes. First, their drug use initiation stories were told to show their reflection on weight loss when they started using drugs. Second, experiences of weight loss after drug consumption were presented. Finally, we analyzed the barriers for these women in terms of achieving abstinence, that is, their concerns about regaining weight.

### 3.1. Drug Use Initiation for Weight Loss

Fourteen female drug users expressed that their initiation to drugs was due to weight loss, regardless of which drug they used. In these women’s views, using drugs was a “convenient” and even “ideal” way to achieve and maintain a slim body shape, and they formed the idea based mainly on their friends’ experiences.

For example, 28-year-old Bao mentioned that she started using heroin as she “heard from friends that using it [heroin] was good for losing weight”, which was exactly what she wanted as she “got fatter after giving birth to the daughter”. “I always felt that I was fat and wanted to lose weight, then my friend introduced me to meth”, a 22-year-old drug user, Peng, similarly shared of her introduction to methamphetamine. She also pointed out that she became thinner immediately after trying the drug.

Such as Bao and Peng, almost all of these 14 women received information that drugs were “helpful in losing weight” from their friends, and mostly close female ones. “The best way to keep fit” (Kang, 43 years old) was the most common notion communicated to participants. Based on friendships, these women accepted such recommendations without careful consideration and verification. In addition to their friends’ verbal introduction and persuasion, some participants even stated that they saw the “magic power” that drugs had on their friends’ body weight and shape. Upon seeing the “successful outcome”, these women believed in the capacity of illicit drugs to control weight even more assuredly. A typical example of this kind of thought process came from a methamphetamine user, Gong:

*I was shocked at a friends’ gathering. You cannot imagine that one of my friends became extremely slim, and all other friends thought she was more attractive than before. You know, she was a very fat girl then, over 90 kg [1 kg equals 2.2 pounds, same hereinafter]! My two friends and I were very curious about how she could lose weight in such a short period of time. To satisfy our curiosity, she took out a bottle and told us how to use meth. She said that meth was her ‘magic medicine’; it was not addictive and could help her lose weight. So I decided to use meth that day. It was fantastic that I quickly lost over 5 kg in a few days! (Gong, 27 years old.)*

With their friends’ introduction and recommendation, and together with “the curiosity of drugs’ miraculous effect” (Ye, 30 years old), these women generally chose to try drugs directly without a thorough deliberation. They had little knowledge of the drug that they would take prior to their usage. “I had no idea what meth was”, stated Xiang, a 24-year-old methamphetamine user. This sentiment was also expressed by several other participants. They disregarded the negative health consequences related to drug use, concentrating simply on “the drug’s effect on weight loss” (Lan, 22 years old).

Another poignant example was a woman in her thirties, Yao, who explained that she had a “huge figure” and “couldn’t fit into any available sizes of beautiful clothes”. Losing weight was the only reason she chose to use methamphetamine, as she failed to achieve weight loss through other methods:

*It was just for losing weight. I worked in a nightclub as the business director. That work was very easy and I had nothing to worry about. Because of my comfortable life, I was getting fatter and fatter. I realized that I had to lose weight. I could not be that fat any longer. I had used several kinds of weight-loss pills and exercises, but nothing helped. One day my friend introduced me to meth as another weight control medicine, and that was how I started meth use. To be honest, I didn’t know that meth was an illicit drug, I only regarded it as a thing to lose weight and it really could help. (Yao, 38 years old.)*

However, unlike Yao who was overweight, the majority of the participants (25 out of 29) had very normal or below normal weights according to the BMI classification, although they mostly thought they were “really fat”. In fact, quite a few women’s (9 out of 29) weight was less than 50 kg on their initiation of drug use. For example, one woman mentioned that she was only 46 kg when she decided to use methamphetamine to lose weight:

*I was 46 kg. Friends all said I was thin, but I still thought I was fat. I wanted to lose weight. One friend told me that using meth was a good way to lose weight, so I tried. It was useful! (Wei, 21 years old.)*

### 3.2. Weight Loss Experiences Due to Drug Use

As these female drug users expected, after taking the drug, they experienced weight loss. The “positive” outcome in turn strengthened these women’s belief that illicit drugs could facilitate weight loss. Fifteen participants’ stories of drug use related to weight loss, and they shared their experiences and attitudes. For example, methamphetamine user Ling (40 years old) stated that “meth can make people lose weight very quickly, and I lost 15 kg in ten days after using it!” Shi even recalled that she was only about 35 kg when she was using heroin before entering the compulsory drug treatment center.

When explaining the reasons for weight loss after drug use, most participants believed that it was due to drugs’ effects in terms of “decreasing appetite” (Kang, 43 years old). One of them stated: “I lost my appetite when I used heroin. I didn’t eat anything and that was why my weight reduced so quickly” (Zhu, 43 years old). “I only had some soft drink, ate nothing, and that was why heroin made me extremely thin”, stated another participant (Hua, 40 years old). Different from the heroin users who addressed appetite loss only, methamphetamine users pointed out that appetite loss and sleep loss were both considered to be influential factors behind weight loss. “Couldn’t sleep and didn’t want to eat”, 26-year-old Mang shared when talking about the consequences of using methamphetamine, as a result of which she “became very thin”. A few polydrug users even compared the differences between heroin and methamphetamine to highlight the importance of sleep deprivation in losing weight.

*When I used meth, I didn’t eat and didn’t sleep. Look at me, I am quite tall, but I was just 43.5 kg at that time. Can you imagine how thin I was? Using meth was a kind of energy overload, more painful than using heroin. Especially the sleep deprivation, almost killed me and also made me extremely thin. (Nan, 31 years old.)*

Similar to Nan, a few other participants admitted that their thinness was “unhealthy” (Lian, 22 years old), “unenergetic” (Qiang, 16 years old), and was associated with “a pale face” (Hua, 40 years old). A heroin user even described that she was “as thin as a ghost” (Juan, 37 years old). Although these female drug users were fully aware that their slim body shape came at the expense of health, the majority of them still “felt happy” (e.g., Ping, 22 years old; Mang, 26 years old) with their weight reduction. This result, thus, confirmed their belief and expectation of illicit drugs’ function in weight control. One interviewee, Lan, clearly revealed that the extreme unhealthy thinness was exactly what she wanted:

*I used meth only for losing weight. When I felt that I was getting fat, I would use it. It helped me to keep slim. Of course it would have some influence on my body. I mean my cheeks might not look rosy, and people might think I was ill. But I could be very, very, very thin, and that was my goal. (Lan, 22 years old.)*

### 3.3. Barriers to Achieving Abstinence: Concerns about Regaining Weight

There were ten participants who expressed that they were not confident in abstaining from drug use, since they were afraid of regaining weight. A typical case was that of Fang, a 19-year-old methamphetamine user:

*I don’t think I’m addicted to meth, but I really need it! I was not that fat, but I wanted to be thinner, so I used meth. I then became thinner and thinner and I was happy with it. In fact, I once tried to get rid of meth. But when I stopped using it, I suddenly became very fat! I was really fat in a flash, which I couldn’t stand! To regain my slim figure, I thought that I had to use meth again. (Fang, 19 years old.)*

In addition to Fang, a few other female drug users also experienced weight gain after ceasing drug use. Most reported that they had gained 15 kg to 25 kg, which they were “unwilling to face” (Ye, 30 years old). “Can you imagine that I had gained 25 kg? Horrible!” Zhong (44 years old) stated when she recalled her previous drug treatment experiences.

In the absence of drugs, “endless eating and sleeping” (Ling, 40 years old) was believed to be the reason for an increased weight and for becoming “abnormally fat” (Zhu, 43 years old). “I ate a lot when I stopped using meth”, stated Ding (19 years old). Another participant, Ming, used her experience to further explain:

*Drugs cause appetite to decrease and cause weight loss. When I used meth, I could go three to four days without eating or sleeping. I could definitely lose weight then. But when I didn’t use the drug, my appetite would be very good and I could eat a lot. I ate almost everything; even the things I didn’t like to eat before were delicious to me. Therefore, my weight increased fast and I became even fatter than I was before I started using meth. (Ming, 36 years old.)*

When they saw that their weight was increasing, these women would simply resume drug consumption, since they believed that “using drugs was the quickest way to lose weight” (Lu, 32 years old). From these women’s perspectives, drug consumption was even believed to be the “only effective method” (Yue, 24 years old) to keep slim. Kang had several rounds of drug treatment, but she relapsed every time because she could not tolerate her weight increasing after treatment; “the only thing I could do was go back to use drugs; first it was heroin, later it was meth” (Kang, 43 years old). As a polydrug user, Yun (38 years old) revealed that, with drugs, she could “get down to a satisfactory weight in just one day!”

## 4. Discussion

In keeping with the findings of prior research in some western societies [17,18], Chinese female participants in this study used drugs as unhealthy weight control tools, to reach and maintain a desired weight and body shape. As seen in previous research [9], these women were all dissatisfied with their figures and constantly planning to lose weight, even though they were already very slim or at least within their healthy weight range. For these women, using illicit drugs to achieve weight loss was a positive experience, even though they had noticed negative health outcomes after drug consumption. Indeed, Chinese women’s belief in illicit drugs to aid them in weight management was a central motivating factor in both their initiation and maintenance of drug use.

In order to quickly lose weight and achieve a desired body shape, these women began drug use through both accepting their friends’ recommendations and witnessing drugs’ “miraculous effect” on their friends. This finding largely confirmed that Chinese women’s drug use behaviors are heavily influenced by “high-risk social networks” to which they belong [24]. Before starting to use drugs, they were, for the most part, unaware of the negative health consequences that drugs might bring. This finding was also consistent with a previous finding that Chinese female drug users had little drug-related knowledge prior to their using [28]. Later, when they experienced ill health from drug consumption, mentioned by several participants in this study, they generally still valued its contribution to their weight loss. When they regained weight during or after the drug treatment process, they chose to relapse without hesitation. Certainly, Chinese female drug users mostly liked using drugs because it helped them to become extremely thin in line with their expectations.

### 4.1. The Pursuit of Thinness and Risk-Taking

Substance use, whether for weight loss or otherwise, is an unhealthy, risk-taking behavior. Risk-taking behaviors are those performed under uncertainty and without robust contingency planning, and these behaviors usually have negative consequences and may cause harm to the individual engaging in them or others [33]. Previous research has found that men generally have higher risk-taking tendencies than women [34], and women appear less likely than men to manifest undesirable related personality traits such as aggressiveness, impulsiveness, and sensation-seeking [35]. Yet, according to the findings of this study, Chinese female drug users were willing to take risks and also sacrifice their health to achieve the goals of weight control. This behavior indicates that women’s pursuit of thinness can be extremely unhealthy indeed [36,37]. Their belief in illicit drugs’ efficacy with respect to weight control may lead them to overlook the potential risks of drug consumption.

The cultural preference for a slim body shape (e.g., thinness implies attractiveness) [38] and the implicit and explicit discrimination against overweight people (e.g., being mocked for being unable to purchase suitable clothes) [20] also contributed to these women using drugs to manage weight. This finding was consistent with studies that show women are more likely to engage in weight control practices under social pressure and social evaluation [12,39]. For the women in this study, successfully aspiring to the ideal of a slim figure brought them pleasure, as it made them attractive according to this cultural norm. Drug use brought them so much pleasure, in fact, that they could overlook the negative impact on their health, which included a pale face and a lack of vigor. Compared with the benefits of thinness, such health implications were very much secondary in these women’s perceptions.

### 4.2. Peer Influence and Differentiated Normalization

According to the findings of this study, using illicit drugs as weight management devices was prevalent within the female drug users’ social network. Experienced drug users were also willing to share information and persuade their friends to use drugs for weight loss purposes. This confirmed the “differentiated normalization” [28,40,41] explanation of drug subcultures. It indicates the normalization of drug use among certain social groups, demonstrating the complexity of people’s perspectives towards drug use [28]. People in these particular social groups have greater likelihoods of using illicit drugs since drug consumption is generally perceived as acceptable or even desirable behavior [28,42,43].

In addition, the findings also indicated that peer influences had a considerable impact on Chinese women’s drug use, which also conformed to previous findings that “having high-risk social networks was a common theme in drug initiation” [24] (p. 399) among Chinese female drug users. More broadly, this peer influence also reflected that, in the Chinese cultural context, individuals tend to trust people within small social circles [44], which markedly reduces actors’ perceptions of the risks involved [45].

### 4.3. Implications

Three major implications were raised based on the findings and discussions of this study. Firstly, the dominant aesthetic preference of thinness needs to be changed. Mainstream media and educational organizations should foster inclusiveness of diverse body types. Only when thinness is not seen as a necessity for a beautiful female body will women renounce unhealthy weight-loss behaviors. Further, even if some women need to lose weight for health reasons, they should be better informed that using illicit drugs is not a wise choice in doing so.

Secondly, the functional use of illicit drugs, such as for losing weight, should also be brought to the forefront during drug treatment. Concern about weight and body shape is likely to be one of the foremost reasons in general for Chinese female drug users’ frequent failures to successfully abstain over the long-term. Treatment programs should better educate drug users that drug rehabilitation could lead to certain consequences (e.g., weight gain). Such a step could prepare the treatment recipients psychologically to deal with changes in a more positive way; thus, decreasing the chances of relapse. In addition, drug treatment centers are also encouraged to incorporate healthy eating and exercise more fully into their rehabilitation work.

Finally, more social work interventions should be provided to women who interact with drug users in their social networks, that is, in light of peer influence regarding the use of drugs. These interventions are suggested to cover the following two aspects: (1) offer these women enough knowledge of the dangers of drug use and the negative impacts of drug use on health; (2) help them learn to look at their bodies in a positive light instead of pursuing unhealthy, extreme thinness. These efforts may be of great help in preventing or reducing the possibility that women use drugs for weight control.

### 4.4. Research Limitations and Future Directions

This study had several limitations, based on which we propose the following five directions for future studies.

Firstly, this study only focused on women, mainly because women are more likely to be affected by ideas about body image and tend to pursue thinness [9,10]. However, the differences between women and men in engaging in risk-taking behaviors for the sake of their body image requires further study.

Secondly, this study was based only on participants’ personal accounts of drugs’ impact on weight loss, focusing on individual views of the utility of drugs in this regard. Future studies might choose multiple methods in measuring and understanding the relationship between drug use and weight control. These methods can include questionnaires, psychological scale measurements, and physiological index measurements [46]. In particular, more information could be collected in future research to show different effects of different drugs.

Thirdly, when considering weight loss and weight control practices, this study only took drug use behavior into consideration, but not other risky or unhealthy behaviors. Therefore, future studies are recommended to explore other behaviors related to weight control and, more broadly, weight management among Chinese women, and also men.

Fourthly, due to the nature of the compulsory drug treatment centers, our participants were all recruited with the help of the center’s administrative officers. Those drug users who were familiar with the administrative officers might have been more likely to be recommended [28]. The officers’ exact selection process for recommending participants remains unclear and, thus, a bias might exist [47]. Future studies should consider this issue and may choose to use community samples to avoid this problem.

Lastly, as an exploratory qualitative study, the sample only included 29 female drug users, which was quite small. The findings of the study, thus, could not be generalized to the greater population of female illicit drug users in China. Future studies, both qualitative and quantitative, are encouraged to cover more participants in order to develop a firmer basis for generalization.

## 5. Conclusions

Based on a selected sample of recovering female drug users in China, the findings of this exploratory study revealed a link between drug use practices and weight control expectations. Although it had several limitations, this study contributes to the existing literature by highlighting the functional role of drug use in Chinese female drug users’ lives. Illicit drug use among Chinese women has caused a number of health problems. China’s ever-broadening illicit drugs market has further aggravated its domestic situation regarding the control of drugs, owing in no small part to the exponentially increasing number of female drug users. Therefore, an understanding of Chinese women’s weight-loss-related drug use patterns contributes to the formulation of necessary strategies and policies to prevent this growing problem.

## Figures and Tables

**Table 1:** Key demographic variables of 29 participants.

Variable		*n* = 29 *n* (%)/M (Range)
Age		31.0 (16–48)
Education level	Primary school and under	4 (13.8%)
	Middle school	13 (44.8%)
	High school (including vocational high school)	10 (34.5%)
	Junior college and above	1 (3.4%)
	Not available	1 (3.4%)
Marital status	Single (including divorced and widowed)	23 (79.3%)
	Married (including cohabitation)	4 (13.8%)
	Not available	2 (6.9%)
Employment status (prior to the treatment)	Legal full-time job	13 (44.8%)
	Sex-related job	7 (24.1%)
	Self-employed or running small business	4 (13.8%)
	Unemployed	5 (17.2%)
Drug used	Heroin	1 (3.4%)
	Methamphetamine	14 (48.3%)
	Poly	14 (48.3%)

## Data Availability

The data presented in this study are available on request from the corresponding author. The data are not publicly available due to the privacy.
